# The performance of single and multi-collector ICP-MS instruments for fast and reliable ^34^S/^32^S isotope ratio measurements†

**DOI:** 10.1039/C6AY02177H

**Published:** 2016-09-23

**Authors:** Ondrej Hanousek, Marion Brunner, Daniel Pröfrock, Johanna Irrgeher, Thomas Prohaska

**Affiliations:** a University of Natural Resources and Life Sciences, Vienna, Department of Chemistry, VIRIS Laboratory, Konrad-Lorenz-Straße 24, 3430 Tulln, Austria; b University of Natural Resources and Life Sciences, Vienna, Department of Forest- and Soil Sciences, Institute of Forest Ecology, Peter-Jordan-Straße 82, 1190 Vienna, Austria; c Department of Marine Bioanalytical Chemistry, Institute of Coastal Research, Helmholtz-Centre for Materials and Coastal Research, Max-Planck-Straße 1, 21502 Geesthacht, Germany

## Abstract

The performance and validation characteristics of different single collector inductively coupled plasma mass spectrometers based on different technical principles (ICP-SFMS, ICP-QMS in reaction and collision modes, and ICP-MS/MS) were evaluated in comparison to the performance of MC ICP-MS for fast and reliable S isotope ratio measurements. The validation included the determination of LOD, BEC, measurement repeatability, within-lab reproducibility and deviation from certified values as well as a study on instrumental isotopic fractionation (IIF) and the calculation of the combined standard measurement uncertainty. Different approaches of correction for IIF applying external intra-elemental IIF correction (aka standard-sample bracketing) using certified S reference materials and internal inter-elemental IIF (aka internal standardization) correction using Si isotope ratios in MC ICP-MS are explained and compared. The resulting combined standard uncertainties of examined ICP-QMS systems were not better than 0.3–0.5% (*u*_c,rel_), which is in general insufficient to differentiate natural S isotope variations. Although the performance of the single collector ICP-SFMS is better (single measurement *u*_c,rel_ = 0.08%), the measurement reproducibility (>0.2%) is the major limit of this system and leaves room for improvement. MC ICP-MS operated in the edge mass resolution mode, applying bracketing for correction of IIF, provided isotope ratio values with the highest quality (relative combined measurement uncertainty: 0.02%; deviation from the certified value: <0.002%).

## 1 Introduction

Natural S consists of four stable isotopes: ^32^S (relative abundance: 94.99%), ^33^S (0.75%), ^34^S (4.25%) and ^36^S (0.01%).^1^ The variation of the relative abundance of the S isotopes in nature is a result of kinetic and thermodynamic effects during *e.g.* Earth’s core–mantle differentiation,^2^ uptake and metabolism of S compounds by fauna and flora^3^ or crystallization and evaporation of seawater.^4^ Analysis of ^33^S/^32^S, ^34^S/^32^S and ^36^S/^32^S isotope ratios reveals processes that cause both mass-dependent and mass-independent isotopic fractionation. The latter is *e.g.* the case of heterogeneous reactions between organic matter and S-bearing aqueous solutions.^5^ It can be applied *e.g.* for the identification of branched reactions and finite reservoir effects.^6^ In the further considerations, however, we focus on the ^34^S/^32^S ratio as the mostly investigated S isotope ratio.

Inductively coupled plasma mass spectrometry (ICP-MS) has become the method of choice for quantitative determination of S at low amounts and within complex matrices.^7^ During the last few years, ICP-MS has also become a powerful alternative to the classical gas source isotope ratio mass spectrometry (IRMS) for S stable isotopes analysis.^6^ The main advantage of ICP-MS over IRMS is the significantly smaller total amount of S required. Thus, isotopic analysis of small constituents of body fluids (cells) or small aliquots of serum collected during blood tests is possible by MC ICP-MS.^6^ Alternatively, small amounts of S (down to 0.7 μmol) dissolved in water can be analyzed with reasonable water sample volume using this technique.^8^ In addition, extended sample preparation steps (conversion of S to SO_2_ or SF_6_ (ref. 2)) can be circumvented when applying ICP-MS. This is in particular of advantage when a large series of natural samples of low volume and/or low S content have to be analyzed.^9^

Spectral interference is the major obstacle for the accurate measurement of sulfur by ICP-MS. Interference on S isotopes^10^ can be resolved either by applying higher mass resolution in sector field mass spectrometers (both single and multi-collector ICP-SFMS instruments^11^,^12^), or by a chemical reaction/interaction of ions with reactive/collision gases in a pressurized multipole cell. Such systems are applied in different commercial ICP-QMS systems. Oxygen is most frequently used as a reactive gas leading to the transformation of S^+^ into SO^+^. Therefore, S isotopes are measured at *m*/*z* 48 (^32^S^16^O^+^) and 50 (^34^S^16^O^+^).^13^,^14^ In an ICP-MS/MS system, one scanning quadrupole is applied in front of the reaction cell and one behind the cell to eliminate a number of concurring molecular interferents.^14^ Alternatively, non-reactive collision gases are used to effectively reduce O_2_^+^ transmission and improve the detected S^+^/O_2_^+^ ratio by means of energy discrimination. Xenon,^15^ or He and Xe^16^ and H_2_, He and Xe^17^ gas mixtures were reported to be suitable collision gases for S measurements. As an additional asset, collisional dumping (aka collisional focusing) leads to a potential improvement of the isotope ratio precision.^18^ In addition, a membrane desolvation unit coupled to ICP-MS reduces the amount of solvent vapor introduced into the plasma, leading to a significant decrease of the interfering oxygen signal. According to some authors, the background signal can be suppressed so effectively that even low mass resolution (*R* ~ 300) can be applied for a precise ^34^S/^32^S measurement in 1–2 μg g^−1^ S solutions.^19^,^20^

The resolving power of the commercially available ICP-MS instrumentation is not high enough to separate ^36^S^+^ from ^36^Ar^+^. Thus, the investigation of mass-independent fractionation is restricted to the analysis of ^33^S only.^8^ However, mass resolution (*m*/*z*/Δ(*m*/*z*)) higher than 4000 must be applied^10^ and the formation of ^32^S^1^H^+^ and ^16^O^16^O^1^H^+^ must be monitored.^8^

Matrix elements can cause non-spectral interference in ^34^S/^32^S analysis as observed for Fe,^10^ Li, K^9^ and recently, in a detailed study, for Ca.^21^ These effects contribute to the instrumental isotopic fractionation (IIF, commonly referred to as instrumental mass bias). The IIF describes the sum of effects in a mass spectrometer that lead to a difference between the detected isotope ratio and the true isotopic composition of the measured element.^22^ Usually, standard-sample bracketing is used to correct for this effect in the case of ^34^S/^32^S measurements.^8^–^10^,^19^,^23^ However, to guarantee the same conditions for the sample and the standard, the matrix composition and the S concentration of both must be equal. Therefore, sulfur–matrix separation is a prerequisite for reliable isotope ratio measurement by ICP-MS. This can be performed *e.g.* by separation of cations using a cation-exchange column,^10^ or a strong anion-exchange resin.^9^ However, the sulfate-S isotope composition can be fractionated when the applied resin becomes saturated. Such fractionation by anion exchange and the enrichment of ^34^S using was even applied to produce compounds enriched in ^34^S.^24^ Thus, the separation procedure has to be validated accordingly for accurate isotopic analysis.

The use of an internal standard represents an alternative, commonly applied strategy. The element used as an internal standard should ideally have the same ionization energy and similar mass to the measurand. Silicone isotope ratios have been applied^12^,^16^,^25^ in the case of S isotope ratio measurements.

The basic different operating principles of single collector and multi-collector ICP-MS instruments are the sequential measurement of multiple isotopes at one detector *vs.* the simultaneous measurement of isotopes at multiple detectors, respectively. The latter is the preferred option for precise isotope ratio analysis. Narrower mass regions (up to single point peak hopping), shorter dwell times and a larger number of measurement cycles are chosen to approximate simultaneous detection and achieve a fast scan between analyzed isotopes in a single collector instrument.^26^ On the other hand, the measurement precision becomes poor when dwell times are set too short.

While dwell times of 10.4 ms and 52.0 ms for *m*/*z* 48 and 50, respectively, were found to be optimal for ICP-QMS,^27^ only 1 ms and 5 ms (for ^32^S and ^34^S, respectively) were chosen in the more sensitive single collector ICP-SFMS.^20^ Besides counting statistics, the measurement precision is affected by sample introduction and plasma fluctuations in the ICP source. The measurement precision of the sample and of the calibrant used for the correction of IIF, the effect of dead time (in the case of secondary electron multipliers used in the pulse counting mode) and the effect of the background have to be taken into account for uncertainty estimation.^28^

The increased use of ICP-MS for S isotope ratio analysis in the last few years was the motivation behind this work in order to demonstrate the performance of more commonly accessible single collector ICP-MS systems in comparison to MC ICP-MS. Limits of detection, measurement precisions, within-lab reproducibility, instrumental isotopic fractionation, combined measurement uncertainty and deviation of the measured from the certified value were evaluated. The IIF effect caused by the acceleration voltage of an ICP-SFMS was demonstrated. For the first time, the ICP-MS/MS was evaluated for the measurement of natural S isotope ratios (although it has been applied for quantitative determination of S, *e.g.*, using isotope dilution^14^).

## 2 Materials and methods

### 2.1 Instrumentation

Five different ICP-MS instruments were used to demonstrate their capabilities for isotope ratio measurements. The measurement strategies (mass resolution, measurement statistics) were optimized for each system individually to cope with spectral interference and instrumental isotopic fractionation and to obtain optimum isotope ratio precision. Detailed information on instrument settings including measurement statistics is provided as ESI S1.†

The MC ICP-MS Nu Plasma HR (Nu Instruments Ltd., Wrexham, UK) is equipped with 12 Faraday cups in a fixed position (three additional secondary electron multipliers were not used in this study). High and edge resolution modes^29^ were applied in this study. As the mass resolution modes are not sufficient for resolving the ^33^S signal from the ^32^S^1^H signal, only the isotopes ^34^S and ^32^S were evaluated. The sample was introduced using a desolvation unit (DSN 100, Nu Instruments Ltd.). The MC ICP-MS operating parameters are summarized in Table 1. The MC ICP-MS was operated in three different measurement modes depending on the strategy for the correction of IIF: a static measurement mode (axial magnet mass was kept constant during the measurement) was applied for S or Si isotope ratio measurements using external correction (bracketing) for IIF. A dynamic method was applied for internal correction of IIF by altering zoom lens voltages (peak alignment and peak shape) and axial magnet mass (*m*/*z* 33 and 29) sequentially (see Table 2) since the used instrumentation does not allow the simultaneous measurement of all masses (28, 29, 30, 32 and 34). Two additional lens voltages (image plane rotation at the collectors and ion beam focus on the low mass collectors) had to be set for measuring mass 28. Finally, a ‘quasi-dynamic’ method was applied: S and Si were measured in three subsequent blocks in a sequence consisting of 6 measurement blocks.

The single collector ICP-SFMS Element 2 (Thermo Fisher Scientific, Waltham, MA, USA) was operated in medium mass resolution (*R* ~ 4000) and E-scan modes only (magnet mass was kept constant). The sulfur concentration was adjusted to reach signal intensities above 100 000 cps at *m*/*z* 32 and 34 to enable the detection in the analogue mode. An APEX-ACM and a cooled (2 °C) cyclonic spray chamber PC^3^ (both from Elemental Scientific Inc., Omaha, NE, USA) were used for sample introduction. The instrumental parameters are given in Table 3 (note: Element XR (analogous instrument) was used at a later stage under the same conditions to confirm the performance of Element 2).

The ICP-QMS NexION 350D (Perkin Elmer, Waltham, MA, USA) was operated in the reaction mode and O_2_ was applied as a reactive gas. The cell parameters (RPq and cell gas flow) were optimized to maximize sensitivity and signal stability at *m*/*z* 48 and 50 in the pulse counting mode. An Aridus II (Teledyne CETAC Technologies, Omaha, NE, USA) and a cooled (2 °C) cyclonic spray chamber (Perkin Elmer) were used in comparison. The measurement parameters are summarized in Table 4.

The ICP-MS/MS Agilent 8800 (Agilent Technologies Inc., Santa Clara, CA, USA) is based on the concept of tandem mass spectrometry. Details on the operation principles of the instrument can be found elsewhere.^14^ In this study, the first mass analyzer was switched between *m*/*z* 32 and 34. Oxygen was used as a reaction gas in the octopole cell. The second mass analyzer was switched between *m*/*z* 48 and 50. An APEX-spiro TMD (Elemental Scientific Inc.) and a cooled (2 °C) double-pass spray chamber (SSI, Elemental Scientific Inc.) were used for sample introduction. The operation parameters are shown in Table 5.

The cell of the ICP-QMS Agilent 7700 (Agilent Technologies Inc.) was pressurized with Xe as the collision gas. Xenon was used as suggested in ref. 17 which reported the S^+^/O_2_^+^ (background) ratio to be improved by a factor of 10 as compared to the use of He as a collision gas. An APEX-spiro TMD and a cooled (2 °C) double-pass spray chamber SSI introduction system were used in comparison. The operating parameters are summarized in Table 6.

### 2.2 Reagents and standards

Laboratory water type I (18 MΩ cm, TKA-GenPure, Niederelbert, Germany) and nitric acid (p.a., Merck, Darmstadt, Germany) were sub-boiled using sub-boiling distillation systems (MLS DuoPur, MLS, Leutkirch im Allgäu, Germany; Milestone Inc., Shelton, CT, USA). Polyethylene flasks and tubes involved in preparation of standard solutions and measurement were double acid washed using 10% (w/w) and 1% (w/w) HNO_3_ and rinsed with laboratory water type I before use. Standards were gravimetrically diluted with 1% (w/w) HNO_3_.

1 mL HNO_3_ (65% (w/w)) and 1 mL HF (48% (w/w), ultrapure, Merck) were used to dissolve 0.5 g of the IRMM-017 (Institute for Reference Materials and Measurements, Geel, Belgium). Approximately 0.1 g of isotopic reference materials IAEA-S-1, IAEA-S-2 and IAEA-S-4 (all International Atomic Energy Agency, Vienna, Austria) were dissolved by microwave assisted digestion (MLS 1200mega, MLS) using 3 mL HNO_3_ and 1 mL H_2_O_2_ (Suprapur, Merck). The digest was diluted with sub-boiled water to approximately 1 mg g^−1^ S (stock solution). A Si single element standard (Merck ICP standard) was used as the internal standard for the correction of IIF. The certified isotopic reference material IAEA-S-2 was used as the bracketing standard (Table 7).

Solid K_2_SO_4_ (p.a., Merck) was dissolved in sub-boiled water to obtain 1 mg g^−1^ S and used for tuning and optimization of the ICP-MS instruments. H_2_SO_4_ (p.a., Merck) was used for the investigation of possible effects of the desolvation unit. NaOH and KOH (both p.a., Merck) were used for H_2_SO_4_ neutralization. The influence of common matrix components (Na, Ca) was investigated by adding Ca and Na single element standards (both Merck ICP standards) at a concentration of 2 mg L^−1^ each to the certified reference material solutions (2 mg L^−1^ S or Si). The concentration of cations was selected to match with concentrations typically found *e.g.* in a natural soil solution.^9^ The effect of Ca and Ti on the measurements of S isotopes at the corresponding SO^+^ masses by ICP-MS/MS and ICP-QMS in the reaction mode was evaluated by adding Ca and Ti single element standards (both Merck ICP standards) to blank and certified reference material solutions.

### 2.3 Instrumental isotopic fractionation (IIF) correction

Standard-sample bracketing^10^ was applied for the measurement of ^34^S/^32^S isotope ratios by all instruments. Additionally for MC ICP-MS, the application of Si isotope ratios for the correction of IIF using Russell’s law^12^,^22^ was investigated. A modified Russell’s law applying a correction for masses (see below) as described *e.g.* in ref. 30 was used for comparison.

The instrumental isotopic fractionation per mass unit (IIFr_el_) expressed as percentage was calculated using eqn (1):

(1) $${\text{IIF}}_{\text{rel}}=\left(\frac{R_{\text{certified}}}{R_{\text{measured,uncorrected}}}-1\right)\times 100/\Delta (m/z)$$

where IIF_rel_ is IIF per mass unit, *R*_certified_ is the certified isotope ratio, and *R*_measured,uncorrected_ is the measured raw ratio (only corrected for the blank).

The correction for masses was accomplished by dividing the fractionation factor of the Si isotope ratio by the fractionation factor obtained from the measurement of the S isotope ratio in certified reference materials. The quotient was then applied in Russell’s equation. This approach has been applied *e.g.* in ref. 30 in Zn isotope analysis.

### 2.4 Validation parameters

The instrument sensitivity was calculated as the blank (2% HNO_3_) corrected maximum peak intensity per 1 ng g^−1^ S using a one-point calibration of a 1000 ng g^−1^ standard solution. The limit of detection (LOD) and the background equivalent concentration (BEC) of S and Si were calculated using the one-point calibration applying eqn (2) and (3).

(2) $$\text{LOD}=3\times s_{\text{blank}}\times \frac{c_{\text{RM}}}{\left(I_{\text{RM}}-I_{\text{blank}}\right)}$$

(3) $$\text{BEC}=\frac{I_{\text{blank}}\times c_{\text{RM}}}{\left(I_{\text{RM}}-I_{\text{blank}}\right)}$$

where *s*_blank_ is the standard deviation of the blank value, *c*_RM_ is the concentration of the reference material, *I*_RM_ is the intensity of the reference material, and *I*_blank_ is the intensity of the blank value.

The measurement precision was used in total combined uncertainty estimation (see below) and was calculated as the standard deviation of a single measurement, applying the measurement statistics given in ESI S1.†

All investigations and calculations of the short-time repeatability are based on 10 to 18 measurements under the same conditions on one day. The results are presented as 1 RSD.

The reproducibility studies were performed on different measurement days, using different sample introduction systems (cyclonic spray chamber, SSI, APEX-ACM, APEX-spiro TCM, Aridus II) and applying different statistical correction methods (with or without outlier correction). Reproducibility values (1 RSD) were calculated from blank and IIF corrected S and Si ratios of certified reference materials.

The uncertainties were estimated according to ref. 31, taking the ^34^S/^32^S measurement precision, IIF correction uncertainty (consisting of the uncertainty of isotope masses,^32^ the uncertainty of certified reference values and the standard deviation of measurements of the bracketing standard or of the Si isotope ratio measurement) and blank correction (standard deviation of blank solution measurements) into account. The uncertainty was calculated for a single measurement applying the best instrument (measurement) precision obtained. The blank contribution was propagated for all measured isotopes.

The proportional deviation of the measured (blank and IIF corrected) value from the certified isotope ratio (IAEA-S-1) was calculated for repeated measurements according to eqn (4).

(4) $$\text{Deviation}\;(\%)\;=\left(\frac{R_{\text{certified}}}{R_{\text{measured,corrected}}}-1\right)\times 100$$

where *R*_certified_ is the certified ratio and *R*_measured,corrected_ is the measured ratio, corrected for IIF.

## 3 Results and Discussion

### 3.1 Influence of membrane desolvation units on acidic SO_4_^2−^ solutions

The certified reference material IAEA-S-4 consists of elemental S, which was oxidized to SO_4_^2−^ during acid microwave digestion. When applying the acidic solution, SO_4_^2−^ was removed together with the solvent by the membrane desolvation units during sample introduction. This effect was also observed for diluted H_2_SO_4_ and is in agreement with the literature for different systems using membrane desolvation,^8^,^19^ except for the MCN-6000 desolvation unit, which applies a different type of membrane.^11^ As a consequence, the dissolved IAEA-S-4 standard solution had to be neutralized before use (using KOH or NaOH). Since the added amount of Na or K is low (at a level of 2 mg L^−1^ Na or K), no significant matrix effect on the measured isotope ratio of the IAEA-S-4 standard was observed. According to Liu *et al*., the magnitude of the matrix effect depends on the absolute concentration of the dissolved cation.^21^ A matrix effect was reported only at substantial concentrations, estimating the threshold of Na and K to be more than 5 mg L^−1^ and 10 mg L^−1^, respectively.^9^

### 3.2 Analytical figures of merit

#### 3.2.1 Sensitivity, LOD, BEC and the matrix effect

The achievable sensitivities of the ICP-MS instruments are shown in Table 8. The results were accomplished at a comparative level. Differences in sensitivity between the different instrument types can be explained by different transmission efficiency, caused by differences in interface construction (*e.g.*, ion deflection by QMS), acceleration voltages and electrostatic lens systems, vacuum systems, measurement strategies (reaction/collision mode) or mass resolution modes applied. The obtained sensitivities are in a typical range for the used instruments. It is evident that the application of the desolvation systems enhanced the sensitivity compared to conventional spray chambers. Additionally, the application of a high performance skimmer cone (Nu Plasma HR) led to a significant sensitivity improvement in the case of the used MC ICP-MS. In this work, however, the high performance skimmer cone was not used due to repetitive orifice blocking when a larger series of samples were measured. This resulted in a continuous change of the sensitivity and the IIF during a measurement series.

The average sensitivity of Si increased by almost a factor of 2 when measured in the presence of S using the MC ICP-MS in the edge resolution mode. van den Boorn *et al.*^33^ observed an increase in the signal intensity of Si isotopes of 60% in the presence of S when using the Neptune MC ICP-MS. As we can ensure an interference-free measurement of Si isotopes, we assume that the signal intensity increase is favored by the low ionization potential of Si (8.15 eV) compared to the ionization potential of S (10.36 eV). García-Poyo *et al.* observed a similar effect (increase of intensity measured by ICP-MS) of the S matrix on As, Se, and Te,^34^ which all have lower ionization energy than S. The authors explain the signal enhancement by an increase in the analyte ion population as a result of charge transfer reactions involving S species in the plasma. In our study, along with the increased sensitivity, a different IIF of Si isotopes was observed in the presence of S as described later.

The LOD and BEC values are summarized in Table 9 and are based on the same blank solutions. The BEC of S in the used sector field instruments was independent of the sample introduction systems or resolution modes applied. The same BEC was found in different types of water (reagent grade type I and sub-boiled water) and nitric acid of concentrations from 1% to 5% (w/w), or when using Ar from different suppliers. The elevated BEC in the MC ICP-MS was neither affected by the use of different gas lines, nor by installation of new interface parts and a new lens system. High resolution (*R* = 3000) measurements showed that the increased BEC was not caused by interference and similar values were obtained for both isotopes. Similar BEC values were observed also by other scientists using this instrument (ref. 19 and oral communication). Therefore, the observed S background results most probably from bleeding of gaskets or tubing. Although the background signal was higher in MC ICP-MS, it was more stable as compared to ICP-SFMS, resulting in a lower BEC, but a higher LOD of the latter instrument. De Wolf *et al.* used HPLC-ICP-QMS in the reaction mode and HPLC-ICP-SFMS for S detection in glutathione and its conjugates.^13^ The authors reported a 10-fold lower LOD when using ICP-SFMS, mainly due to the ^36^Ar^12^C^+^ signal that cannot be separated when using the QMS instrument. In the presented work, where standard solutions without an organic matrix were analyzed, the LOD obtained for ICP-QMS was approximately 4 times lower than that of ICP-SFMS.

The ICP-MS/MS represents the quadrupole-based instrument of choice, when a low LOD of S is targeted. The obtained LOD was even lower than the results observed *e.g.* in ref. 14. The S BEC was lowest under all investigated instruments. The LOD and the BEC were comparable for both analyzed S isotopes measured at *m*/*z* 48 and 50.

Xenon was reported to be a suitable collision gas for the measurement of S applying an octopole collision cell.^15^ The background signal at the *m*/*z* 32 and 34 (caused mainly by ^16^O^16^O^+^, resp. ^16^O^18^O^+^) was indeed reduced significantly (by a factor of 150 at *m*/*z* 34), leading to a low S BEC (6 ng g^−1^) as compared to ICP-QMS in the reaction mode (14 ng g^−1^) or ICP-SFMS (9 ng g^−1^) instruments.

In the case of the ICP-QMS in the reaction mode, an elevated BEC was observed at a certain point only at *m*/*z* 50 (*i.e.*, ^34^S^16^O^+^). This was caused by the ^14^N^1^H^35^Cl^+^ interference, a result of residual Cl in the instrument (after measuring Cl containing matrices). The BEC accounted for 240 ng g^−1^ S at mass 50 compared to 10 ng g^−1^ at mass 48 (^32^S^16^O^+^). This fact has to be taken into consideration for S isotope ratio analysis in Cl-containing matrices (*e.g.* environmental samples). The same applies for S quantification by means of online isotope dilution using Cl containing buffers.

Balcaen *et al.*^14^ demonstrated the strength of the ICP-MS/MS technique by analyzing S in a simulated matrix containing 50 μg L^−1^ Ca and Ti. It was shown that the introduced interfering ions are separated completely in the MS/MS mode. In this study, the effect of the simulated matrix on the LOD and BEC values was investigated by addition of 1 mg L^−1^ Ca and Ti to the blank solution. This concentration corresponds to levels that can be found in natural samples, *e.g.* for Ca in soil solution.^9^ In comparison, ICP-QMS (using a reaction cell without a mass filter in front of the cell) measurements of the simulated matrix led to an increase of LOD and BEC from 0.2 to 28 ng g^−1^ S and from 16 to 630 ng g^−1^ S, respectively, whereas no significant change of LOD and BEC was observed in ICP-MS/MS.

Based on the presented results, 250 ng g^−1^ S (ICP-MS/MS), 1 μg g^−1^ S (ICP-SFMS and ICP-QMS in reaction and collision modes) and 2 μg g^−1^ S (MC ICP-MS) were chosen as optimum S mass fractions for precise isotope ratio measurements.

#### 3.2.2 Short-time repeatability (precision) and within-lab reproducibility

**Fig. 1:**
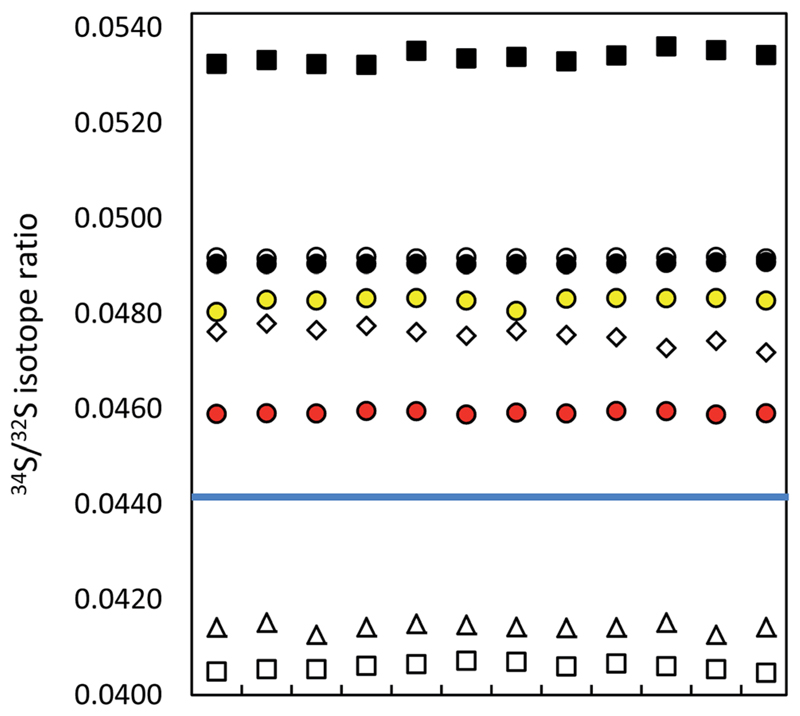
Precision of ICP-MS instruments (12 uncorrected measurements in a series; IAEA-S-1 (blue line)): MC ICP-MS (a) in HR (black circles), (b) in eR (i) applying bracketing (IAEA-S-2; open circles), (ii) in dynamic mode (red circles), (iii) in quasi-dynamic mode (yellow circles), ICP-SF-MS (triangles), ICP-QMS in reaction mode (open squares), ICP-MS/MS (diamonds), ICP-QMS in collision mode (black squares). See also ESI S2.†

The measurement precision of the different instrumentation is displayed in Fig. 1 and summarized in a table form in ESI S2.† As expected, the lowest precision values were obtained by MC ICP-MS in the static mode, due to the fixed magnet field strength (in contrast to the (quasi-)dynamic mode) and the simultaneous detection of S isotopes (in contrast to QMS and SFMS). The difference between measurement precision of static, dynamic and quasi-dynamic modes of the used MC ICP-MS can be explained by the axial magnet mass switch, the zoom lens voltage switching and the additional lens voltage changes needed to focus the mass 28 beam into the lowest mass cup in the (quasi-)dynamic mode (see Table 1). At the time of the experiment, the Nu Plasma software did not allow for an automatic dynamic adjustment within one batch run. Therefore, the lens voltages were applied for both S and Si isotopes in quasi-dynamic measurements. Authors using Neptune MC ICP-MS (Thermo Fisher Scientific) were able to measure Si and S isotopes simultaneously.^16^,^25^ This can be achieved with the new generations of the Nu Plasma, the Nu Plasma II and III, as well (oral communication).

The difference in precision values between the edge mass resolution and high resolution modes can be explained by the peak shape: the measurements in the HR mode (triangular peak shape) are more influenced even by small signal fluctuations. The obtained measurement repeatability was significantly higher than literature values, usually between 0.02% (2 SD)^9^,^35^ and 0.05% (SD),^36^ alternatively 0.01% (standard error).^37^ The best measurement precision of Si isotopes was achieved for the ratio of the most abundant isotopes ^29^Si/^28^Si, no matter in which operation mode (see ESI S2†).

Lower measurement precision of the other instruments was caused mainly by signal instabilities generated by the ICP source, which cannot be cleared by sequential mass analyzers. In the ICP-SFMS, the triangular peak shape in the medium resolution mode limited the precision in the same way as observed by the MC ICP-MS operated at full high resolution. In both cases, the stability of the mass calibration is crucial as even small mass drifts result in a significant change of the measured isotope ratio. Prohaska *et al.*^11^ achieved a significantly lower measurement precision (0.04%) when analyzing a standard solution of only 100 μg L^−1^ S using the first generation of Thermo Scientific Element ICP-SFMS after optimization and substantial thermal stabilization of the whole instrument (which is not reported in detail in the corresponding literature), providing stable measurement conditions at unaltered instrumental settings. Moreover, the authors achieved significantly lower blank levels by operating under class 1000 clean room conditions (which is not reported in detail neither). The second generation of the Thermo Scientific ICP-SFMS instrument applies the ‘lock mass’ feature to cope with thermal instability and the resulting shift. The lock mass correction is performed by the alteration of acceleration voltages and thus shifting the peak back to its original position. A change in the acceleration voltage, however, leads to a change in the observed instrumental isotopic fractionation within the instrument and higher variation of measured isotope ratios within a measurement series (see below). Moreover, since the drift does not influence both masses (32 and 34) to the same extent, the automatic correction leads to deteriorated measurement precisions. Switching the ‘lock mass’ feature off did not improve the measurement precision. In contrast, due to a continuous peak shifting, S isotope analysis was not reproducible (and thus not evaluated). It is reported that the measurement precision can be improved (^34^S/^32^S ratio RSD ~ 0.01% for 100 ng g^−1^ S) by the use of a built-for-purpose slit system for Element 2 and Element XR.^38^ The exit slit width is wider than the entrance slit in this system, resulting in a flat top peak shape at a mass resolution of 2500 providing a solution to the mentioned problem. In the current work, the combination of a high resolution entrance slit with a medium resolution exit slit was tested in the standard system to create flat top peaks. No improvement of the isotope ratio precision could be observed. ICP-SFMS and MC ICP-MS were also compared for S isotope ratio measurement in ref. 37 and ref. 36. San Blas *et al.* obtained measurement precisions of 0.01%, 0.1% and 0.4% when applying Faraday cups and ion counters in MC ICP-MS and using single collector ICP-MS, respectively.^37^ Giner Martinez-Sierra *et al.* observed 4-times higher measurement precision when comparing MC ICP-MS (0.05%) with ICP-SFMS (0.2%).^36^

**Fig. 2:**
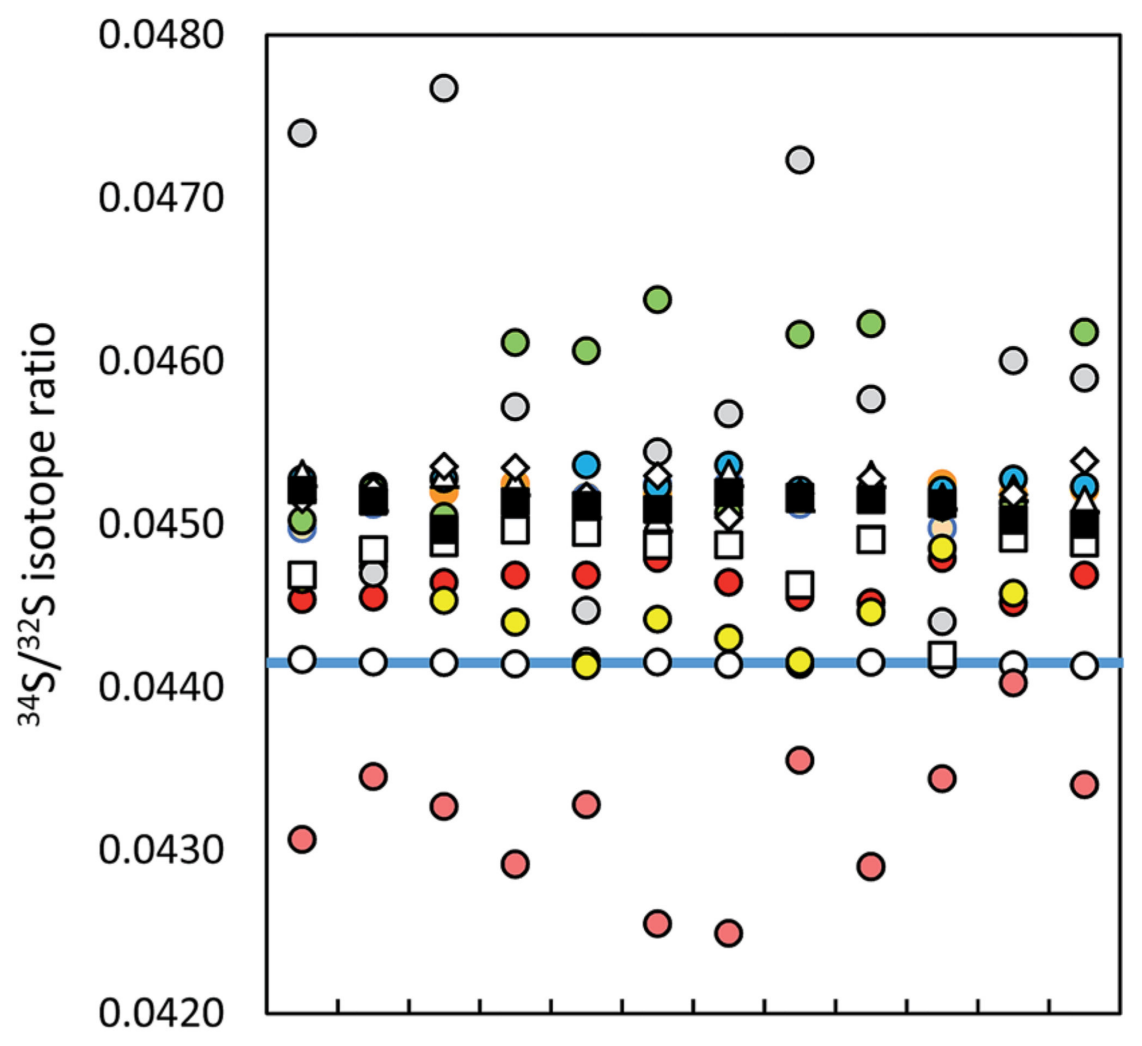
Within-lab reproducibility of ICP-MS ^34^S/^32^S isotope ratio measurements (12 single measurements, IAEA-S-1 (blue line)): MC ICP-MS in eR applying (a) bracketing (IAEA-S-2; open circles), (b) correction by (i) ^30^Si/^28^Si ratio in dynamic mode (red circles), (ii) ^29^Si/^28^Si ratio in dynamic mode (orange circles), (iii) ^30^Si/^28^Si ratio in dynamic mode using HP cones (pink circles), (iv) ^29^Si/^28^Si ratio in dynamic mode using HP cones (brown circles), (v) ^30^Si/^28^Si ratio in quasi-dynamic mode (yellow circles), (vi) ^30^Si/^29^Si ratio in quasi-dynamic mode (blue circles), (vii) ^30^Si/^28^Si ratio in quasi-dynamic mode using HP cones (grey circles), (viii) ^30^Si/^29^Si ratio in quasi-dynamic mode using HP cones (green circles), ICP-SF-MS in MR (triangles), ICP-QMS in reaction mode (open squares), ICP-MS/MS (diamonds) and ICP-QMS in collision mode (black squares). See also ESI S2.†

No significant difference in the measurement precision was observed between the different ICP-QMS instruments. However, the reaction or collision mode do perform better than a direct measurement of SO^+^ signals as performed by Menegario *et al.*^27^ (see ESI S2†). The use of a pressurized cell has also the potential to improve isotope ratio precisions due to the effect of collisional focusing.^18^ The factors limiting a precise isotope ratio measurement using quadrupole instruments are counting statistics and the arrival time distribution – when using the pressurized cell. The arrival time distribution depends on the pressure in the cell, the cell gas flow rate, the cell rod offset potential, the entrance and exit aperture lens potential and the Matthieu parameter RPq. The arrival time had to be optimized for S isotope analysis (by adjusting the settling time *versus* the standard deviation of the measured signal). The within-lab reproducibility for the different instrumentation is shown in Fig. 2. A complete numerical overview is provided in ESI S2.†

The MC ICP-MS operated at edge mass resolution delivers the best reproducibility values, comparable with values reported for other MC ICP-MS instruments.^8^,^10^,^25^ The best reproducibility of Si isotopes was achieved for the ^29^Si/^28^Si ratio, measured at edge resolution in the dynamic mode (see ESI S2†). The reproducibility observed for the single collector ICP-SFMS is mainly caused by the instability of the mass calibration. The mass offset correction by lock mass influences the reproducibility in an analogue way to what was described above for short-time repeatability.

Note that the presented precision and within-lab reproducibility values were obtained by measurement of pure standard solutions. As sulfur–matrix separation is required for the analysis of natural samples (unless matrix-matching of standards is applicable^23^), the method-related isotopic fractionation can influence these analytical figures of merit. The ^34^S/^32^S measurement precision obtained by Hanousek *et al.* for standards purified by an anion exchange resin membrane was low (0.01%) as compared to this study (0.003%) although the same instrumentation and method (MC ICP-MS, eR, bracketing) were applied.^9^ On the other hand, Craddock *et al.* reported a reproducibility value of 0.02% when using a cation exchange resin for sulfur–matrix separation and MC ICP-MS in eR for ^34^S/^32^S analysis.^10^ This value is comparable with the reproducibility obtained in this study.

#### 3.2.3 Instrumental isotopic fractionation per mass unit

**Fig. 3:**
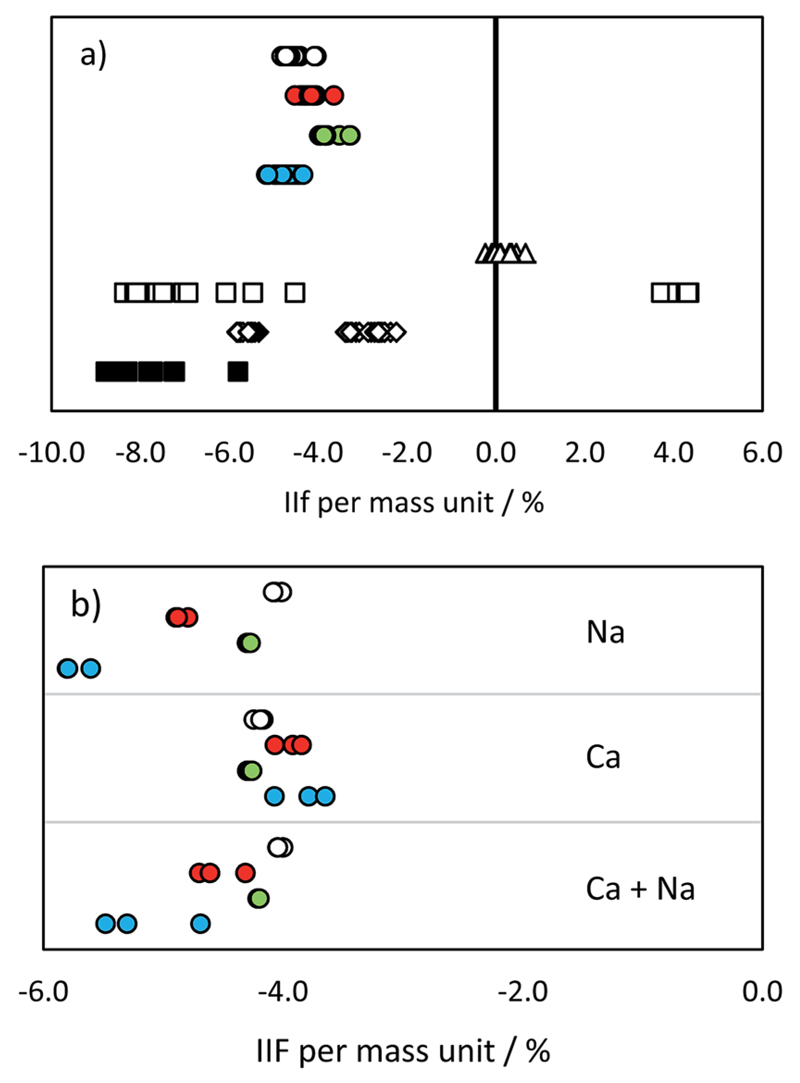
IIF per mass unit (%) in (a) pure IAEA-S-1 solution and (b) IAEA-S-1 solution with added cations: MC ICP-MS ^34^S/^32^S in eR (open circles), MC ICP-MS ^30^Si/^28^Si in eR (red circles), MC ICP-MS ^29^Si/^28^Si in eR (green circles), MC ICP-MS ^30^Si/^29^Si in eR (blue circles), ICP-SF-MS ^34^S/^32^S in MR (triangles), ICP-QMS in reaction mode (open squares), ICP-MS/MS (diamonds), ICP-QMS in collision mode (black squares). See also ESI S2.†

The instrumental isotopic fractionation (IIF) per mass unit is displayed in Fig. 3 and summarized in a table form in ESI S2.†

^34^S/^32^S analysis was accomplished by changing the acceleration voltage while the magnet mass was kept constant (so called E-scan) when using the single collector ICP-SFMS. The variation of the acceleration voltage results in an additional instrumental isotopic fractionation effect, as a consequence of ‘Liouville’s theorem’.^39^ This effect is inversely proportional to the IIF effect caused by the repelling of ions. Therefore, the observed total IIF was small as compared to multi collector measurements. The variation in IIF during the measurement was, again, caused by the lock mass correction of the mass drift. This effect was studied in more detail by changing the magnet mass from 32 to the magnet mass 30 while measuring masses 32 and 34. As a consequence, the acceleration voltage had to be adapted. The resulting IIF per mass unit showed significant differences: 0.23% when the magnet mass was set at mass 32, and −0.32% when the magnet mass was set at mass 30. The standard-sample bracketing has the potential to correct for this effect, but only when the same measurement conditions for the sample and standard are achieved (*i.e.*, the voltage is not changed between samples and bracketing standards) and the mass calibration is not affected during the whole measurement sequence.

The IIF in quadrupole instruments differed from that in sector field instruments due to different extraction potentials and ion optics used. Moreover, according to ref. 16, the use of a collision/reaction cell significantly influences the IIF due to the variation in the transmission rate of residual polyatomic ions. The combination of these factors led to higher IIF per mass unit values and a broader range as compared to SFMS instruments.

The values obtained by the used MC ICP-MS instrument were comparable with the range of 4.0–5.1% reported *e.g.* in ref. 10. Although no significant differences were observed in pure standard solutions (with the exception of IIF of the ^29^Si/^28^Si ratio and ^34^S/^32^S ratio; *p* = 0.05), the presence of Na, Ca and Na + Ca cations influenced the measurement of Si and S isotopes in a significantly different way (Fig. 3b and ESI S2†). The added cations did not cause any significant change in the IIF per mass unit of the ^34^S/^32^S ratio measurement. In contrast, the averaged IIF per mass unit increased by 0.8–1.0% when Ca and Na were added to a Si standard. The threshold of matrix component concentration for accurate Si and S isotope ratio measurement is, therefore, not the same. This effect has to be monitored and taken into account when considering Si isotopes for correcting IIF of S. Our observations are different from the findings of ref. 25 and ref. 12, where a different instrumentation (Thermo Scientific instrument Neptune) was used and ^34^S/^32^S and ^30^Si/^29^Si ratios were measured simultaneously. However, *e.g.* Mason^16^ showed that contrasting ionization efficiencies and chemical properties of S and Si hinder the application of Si isotopes for an accurate IIF correction in MC ICP-MS measurements. The change in plasma conditions and/or in the aerosol generation and distribution as a consequence of S present in the sample were suggested to be responsible for offsets in Si isotope ratios also by ref. 33.

In the case of MC ICP-MS, the applied mass resolution (eR) value was 2700, not sufficient to resolve the ^33^S from the ^32^S^1^H signals. Therefore, only ^34^S and ^32^S isotopes could be evaluated and no conclusions on mass-independence of the fractionation could be drawn. In contrast, Paris *et al.* applied a mass resolution of 8000–10 000 for S isotopic analysis by MC ICP-MS and evaluated ^34^S/^32^S ratios and ^33^S/^32^S ratios in subsequent measurement runs. In this way, a mass-dependent drift of IIF was implicated to be the source of variability between the measurement results.

The mass resolution of ICP-SFMS (4000) was enough to measure an interference-free ^33^S signal. However, as the measurement precision of the ICP-SFMS is an order of magnitude worse than that of MC ICP-MS, the informative value of the measured ^33^S/^32^S ratio was not sufficient to draw further conclusions. Moreover, it can be assumed that the measurement precision (and within-lab reproducibility) deteriorates when additional isotopes are measured. For the same reason, the ICP-QMS instrumentation does not represent a suitable tool for the investigation of the mass-independence of IIF.

#### 3.2.4 Combined standard uncertainty budgets

The results for the combined standard uncertainties are given in Table 10. It is evident that the lowest total combined measurement uncertainty (0.02%, *k* = 1) was achieved by MC ICP-MS in the static mode, applying the edge resolution mode and standard-sample bracketing. The measurement uncertainty of the bracketing standard was mainly (to more than 80%) affected by the uncertainty of the certified value. Thus, the uncertainty of the bracketing standard (column ‘correction for IIF’ in Table 10) could not be reduced by further improvements in measurement precision and it contributed to the total uncertainty by 36.8%. The uncertainty of the reference value does not need to be taken into account if isotope ratios are expressed as delta values (except for the homogeneity of the reference material, which is, however, usually hidden behind the measurement precision of the instrument used for homogeneity studies). Thus, the use of a delta scale can represent a simple way to reduce the combined measurement uncertainty.

The influence of the S blank on S isotope ratio measurement was investigated by estimating uncertainties for different S mass fractions (from 100 ng g^−1^ to 2 μg g^−1^ S). The uncertainty contribution of the blank decreased with the increasing signal to blank ratio from more than 99% (100 ng g^−1^ S) to about 47% (2 μg g^−1^ S). Yet, the correction for the blank remained the major contributor to the total combined uncertainty. Han *et al.* reported comparable *u*_c,rel_ values using the same measurement system in low resolution. Interestingly, the main source of the reported total combined uncertainty (0.04%, *k* = 2) was not the blank, but the measurement precision, although the blank signal contributed to the S (2 mg L^−1^) signal by 5% (ref. 19) compared to a 2% contribution in the presented study. Other reported expanded combined uncertainty values (0.015%,^9^ 0.02%,^12^ or 0.04% (ref. 19)) of ^34^S/^32^S measurements by MC ICP-MS were comparable with results of this study. The internal correction applying the commonly used ^30^Si/^29^Si ratio was burdened with the highest uncertainty (up to 3.6% in the dynamic and 2.6% in the quasi-dynamic mode). The major contributor to these high uncertainty values is the blank correction (up to 99.9%).

The main contributor to the total combined uncertainty of single collector ICP-SFMS was the precision of the isotope ratio measurement. Also here, the limiting parameter can be assumed to be the stability of the mass calibration. The contribution of the blank correction for a 1 μg g^−1^ S standard solution represented only 4% of the total combined uncertainty by ICP-SFMS. The resulting combined standard uncertainty was comparable for all ICP-QMS instrument types. In all cases, blank, IIF correction and the precision of the measured ratio contributed almost equally to the resulting combined standard uncertainty.

#### 3.2.5 Deviation from the certified value

**Fig. 4:**
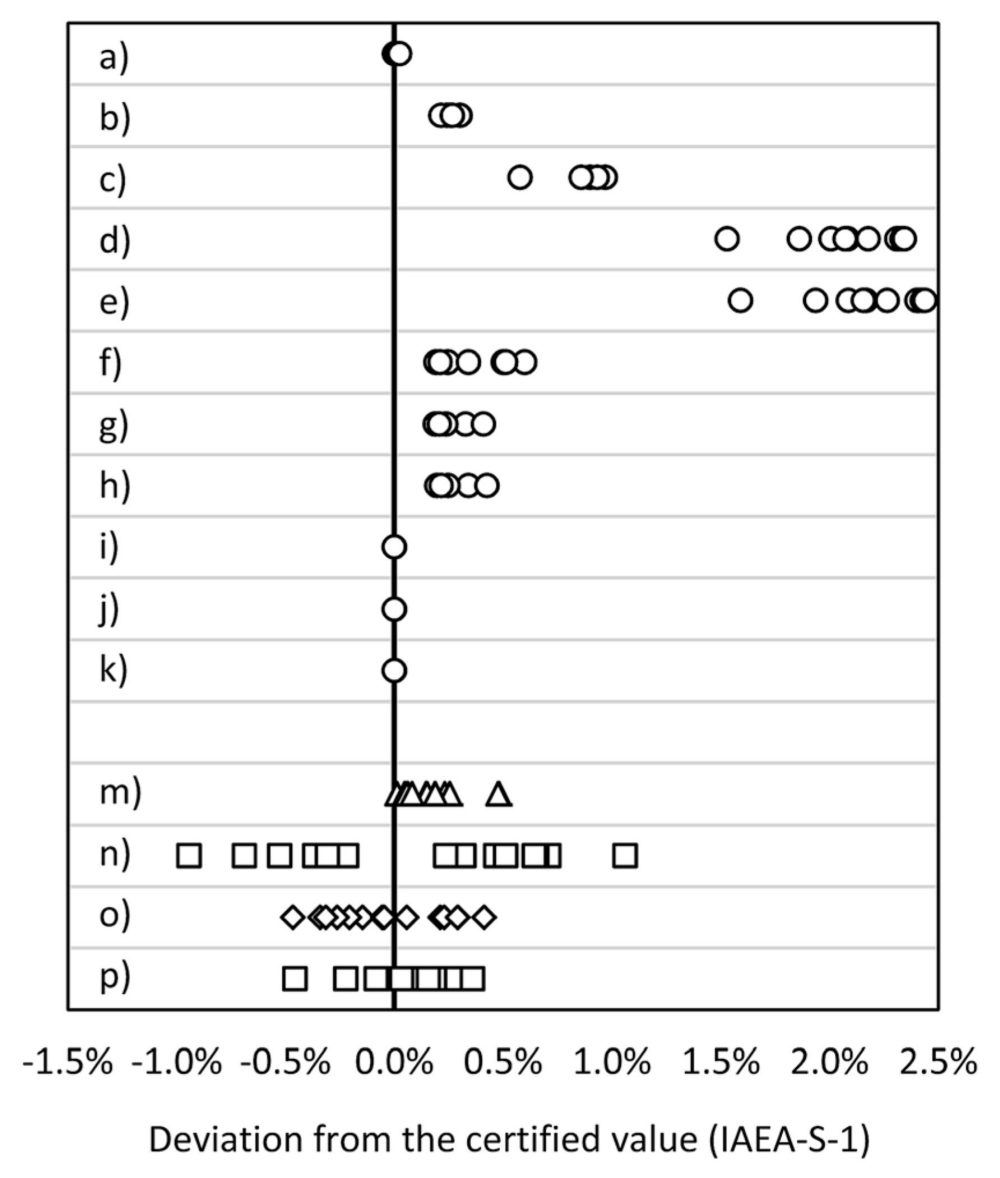
Deviation from the certified value (IAEA-S-1): MC ICP-MS applying bracketing (IAEA-S-2) (a) in eR (*n* = 15), (b) in HR (*n* = 15); MC ICP-MS in dynamic mode applying (c) ^30^Si/^28^Si for internal correction (*n* = 10), (d) ^29^Si/^28^Si for internal correction (*n* = 10), (e) ^30^Si/^29^Si for internal correction (*n* = 10); MC ICP-MS in quasi-dynamic mode applying (f) ^30^Si/^28^Si for internal correction (*n* = 10), (g) ^29^Si/^28^Si for internal correction (*n* = 10), (h) ^30^Si/^29^Si for internal correction (*n* = 10); MC ICP-MS in quasi-dynamic mode applying correction of masses and (i) ^30^Si/^28^Si for internal correction (*n* = 10), (j) ^29^Si/^28^Si for internal correction (*n* = 10), (k) ^30^Si/^29^Si for internal correction (*n* = 10); (m) ICP-SF-MS in MR (*n* = 15); (n) ICP-QMS in reaction mode (*n* = 15); (o) ICP-MS/MS (*n* = 15); (p) ICP-QMS in collision mode (*n* = 15). See also ESI S2.†

The deviation of the obtained ratios from the certified value (IAEA-S-1) is displayed in Fig. 4 and summarized in a table form in ESI S2.† The MC ICP-MS operated in the edge mass resolution mode proved to be most suitable for S isotope ratio measurements. The application of standard-sample bracketing delivers the smallest deviation from the certified value (<0.002%). Correction of IIF by Si isotopes required a correction for masses in order to reduce the deviation from the certified value. Single collector ICP-SFMS as well as ICP-QMS results overlap within corresponding uncertainty (*k* = 1) with the certified range.

## 4 Conclusions

The comprehensive evaluation of the performance of single collector ICP-MS for accurate S isotope ratios showed clearly the still existing limitations. Even novel developments do not provide a substantial alternative to MC ICP-MS. The performance of MC ICP-MS is independent of the IIF correction approach. Internal correction of IIF has the advantage to reduce measurement time if high sample throughput is required. Moreover, possible matrix effects on the measurement are corrected. However, the potentially different instrumental fractionation of S and Si isotopes within a highly variable matrix has to be taken into account when considering a versatile application of the internal correction of IIF. Application of high mass resolution (*R* > 4000) to S isotopic analysis by MC ICP-MS would enable investigation of mass-independence of IIF by additional evaluation of the measured ^33^S/^32^S ratio.

The potential of application of the single collector ICP-SFMS and ICP-QMS instruments for ^34^S/^32^S analysis of natural variations is limited, but by far sufficient *e.g.* for offline or online isotope dilution measurements. Nonetheless, single collector ICP-SFMS still provides substantial room for improvement if modifications are considered, especially with respect to the stability of mass calibration.

One crucial parameter which influences the measurement results and limits possible applications of natural samples (*e.g.* ice cores) is the observed background level of S. This has to be considered even more when sample preparation steps using different chemicals are involved. Instrument modifications might be required, as well, in order to reduce bleeding of gaskets and tubing.

## Supplementary Material

^†^Electronic supplementary information (ESI) available. See DOI: 10.1039/c6ay02177h

Electronic supplementary information

## Figures and Tables

**Table 1:** Operating parameters of the MC ICP-MS (Nu Plasma HR)

RF power	1300 W
Auxiliary gas flow rate	0.75 L min^−1^
Cool gas flow rate	13.0 L min^−1^
Resolution mode	eR (edge mass resolution; *m*/Δ*m* = ~2700)*a*
	HR (high mass resolution; *m*/Δ*m* = ~3000)
*Sample introduction system*	*DSN 100 with a PFA nebulizer*
Nebulizer pressure	30–40 psi
Hot gas flow rate	0.08 L min^−1^
Membrane gas flow rate	3–4 L min^−1^

a Calculated according to ref. 40.

**Table 2:** Faraday cup configuration of the MC ICP-MS for S and Si (nominal masses)

Cycle 1: S		Cycle 2: Si	
Cup	*m*/*z*	Cup	*m*/*z*
Axial	33	Axial	29
L4	32	L5	28
H5	34	H6	30

**Table 3:** Operating parameters of ICP-SFMS (Element 2)

RF power	1300 W
Sample gas flow rate	1.0–1.1 L min^−1^
Auxiliary gas flow rate	1.12 L min^−1^
Cool gas flow rate	16.0 L min^−1^
Sample time	5 ms (^32^S) and 10 ms (^34^S)
Mass resolution	*m*/Δ*m* = 4000
*Sample introduction system*	*APEX-ACM, cooled cyclonic spray chamber*
Sweep gas flow rate	2–3 bar

**Table 4:** Operating parameters of ICP-QMS in the reaction mode (NexION 350D)

RF power	1300 W
Nebulizer gas flow rate	0.92–0.94 L min^−1^
Auxiliary gas flow rate	0.75 L min^−1^
Cool gas flow rate	15 L min^−1^
Dwell time per amu	50 ms (^32^S^16^O^+^) and 200 ms (^34^S^16^O^+^)
Dead time	35 ns
Cell gas flow rate	0.8–0.85 mL min^−1^ O_2_
RPq	0.40–0.50
*Sample introduction system*	*Aridus II, cooled cyclonic spray chamber*
N_2_ gas flow rate	4–5 mL min^−1^
Ar sweep gas flow rate	3–5 L min^−1^

**Table 5:** Operating parameters of ICP-MS/MS (Agilent 8800)

RF power	1550 W
Carrier gas flow rate	1.09–1.18 L min^−1^
Auxiliary gas flow rate	0.89 L min^−1^
Cool gas flow rate	15 L min^−1^
Integration time	50 ms (^32^S^16^O^+^) and 200 ms (^34^S^16^O^+^)
Wait time offset	2 ms
Dead time	31 ns
Cell gas flow rate	0.30 mL min^−1^ O_2_
*Sample introduction system*	*APEX-spiro TMD, cooled double-pass spray chamber*
Sweep gas flow rate	1.6–1.7 L min^−1^

**Table 6:** Operating parameters of ICP-QMS in the collision mode (Agilent 7700)

RF power	1600 W
Carrier gas flow rate	1.25 L min^−1^
Auxiliary gas flow rate	0.9 L min^−1^
Cool gas flow rate	15 L min^−1^
Integration time	50 ms (^32^S) and 200 ms (^34^S)
Dead time	32 ns
Cell gas flow rate	0.10 mL min^−1^ Xe
*Sample introduction system*	*APEX-spiro TMD, cooled double-pass spray chamber*
Sweep gas flow rate	1.8 L min^−1^

**Table 7:** Sulfur and silicon isotope ratio (certified) reference materials. The uncertainty of the Si ICP standard corresponds to SD; the ^30^Si/^28^Si and ^29^Si/^28^Si ratios are expressed with the certified uncertainty (IRMM-017) of the last two displayed digits

Reference material	Material	*δ*(^34^S/^32^S)_VCDT_^a^	^30^Si/^28^Si and ^29^Si/^28^ Si isotope ratio	Other literature *δ*(^34^S/^32^S)_VCDT_ values	
IAEA-S-1	Solid Ag_2_S	–0.30‰ ± 0.00‰		–0.30‰ ± 0.12‰	41
				–0.30‰ ± 0.30‰	25
IAEA-S-2	Solid Ag_2_S	22.67‰ ± 0.20‰		22.67‰ ± 0.09‰	41
IAEA-S-4	Pure S	16.90‰ ± 0.20‰		16.90‰ ± 0.12‰	41
				16.00‰ ± 0.30‰	25
IRMM-017	Pure Si	—	^30^Si/^28^Si: 0.0334889 (78)
			^29^Si/^28^Si: 0.0507715 (66)	
			^30^Si/^29^Si: 0.65960 (19)	
Si ICP standard	SiO_2_	—	^30^Si/^28^Si: 0.03318 (23)^b^
			^29^Si/^28^Si: 0.0504792 (97)^b^	
			^30^Si/^29^Si: 0.6569 (44)^b^	

a Values applied throughout this work.

b Determined *via* IRMM-017 (SD of *n* = 10 measurements).

**Table 8:** Comparison of sensitivity for S (mV or cps per ng g^−1^ S) obtained by different ICP-MS instruments using different sample introduction systems

	Instrument sensitivity/cps per ng g^−1^ S		
Instrument	Desolvation membrane unit		Conventional spray chamber
MC ICP-MS	3 mV (~1.9 x 10^5^ cps)	(DSN 100)	n.a.
ICP-SFMS	10 000	(APEX-ACM)	4600
ICP-QMS (reaction mode)	1150	(Aridus II)	860
ICP-MS/MS	1500	(APEX-spiro TCM)	430
ICP-QMS (collision mode)	1190	(APEX-spiro TCM)	500

**Table 9:** Comparison of limits of detection (LOD) and background equivalent concentrations (BEC) of S and Si (for IIF correction) by different ICP-MS instruments

Instrument	Element	LOD/(ng g^−1^) (this work)	LOD/(ng g^−1^) (literature)	BEC/(ng g^−1^) (this work)	BEC/(ng g^−1^) (literature)
MC ICP-MS	S	0.1	4–38 (ref. 37)	40–50	100,^19^ <10 (ref. 10)
	Si	0.1		5	
ICP-SFMS	S	0.5	0.01 (ref. 11)	9	<10 (ref. 20)
			1 (ref. 13)		
			20 (ref. 42)		
			23 (ref. 37)		
ICP-QMS	S		4 (ref. 43)		
ICP-QMS (reaction mode)	S	0.2	10 (ref. 13)	14	
ICP-MS/MS	S	0.2	0.5 (ref. 14)	3	11 (ref. 14)
ICP-QMS (collision mode)	S	0.3	1.3 (ref. 15)	6	1000 (ref. 17)
			20 (ref. 17)		

**Table 10:** Relative total combined uncertainty (*u*_c,rel_, %) of ^34^S/^32^S isotope ratio measurements (IAEA-S-1, using IAEA-S-2 as the bracketing standard). The contribution of blank, IIF correction and measurement precision to the *u*_c_ is expressed in percentage

Instruments/operation mode	*u*_c,rel_ (*k* = 1)	Blank correction	Correction for IIF	Measurement precision
**MC ICP-MS**				
**Bracketing**				
*Static measurement mode*				
eR	0.02	46.8	36.8	16.4
HR	0.05	0.2	4.9	94.9
**Internal IIF correction – no correction of masses**				
*Dynamic measurement mode*				
Corrected with ^30^Si/^28^Si	0.19	72.5	21.9	5.6
Corrected with ^29^Si/^28^Si	0.09	2.4	74.0	23.6
Corrected with ^30^Si/^29^Si	3.09	99.9	0.1	0.0
*Quasi-dynamic measurement mode*				
Corrected with ^30^Si/^28^Si	0.12	26.0	60.7	13.2
Corrected with ^29^Si/^28^Si	0.10	23.4	60.5	16.1
Corrected with ^30^Si/^29^Si	1.24	87.3	12.6	0.1
**Internal IIF correction – correction of masses**				
*Dynamic measurement mode*				
Corrected with ^30^Si/^28^Si	0.27	39.0	58.0^a^	3.0
Corrected with ^29^Si/^28^Si	0.14	1.1	87.5^b^	11.3
Corrected with ^30^Si/^29^Si	3.62	76.7	23.3	0.0
*Quasi-dynamic measurement mode*				
Corrected with ^30^Si/^28^Si	0.16	10.0	83.2^a^	6.8
Corrected with ^29^Si/^28^Si	0.13	14.4	75.8^b^	9.8
Corrected with ^30^Si/^29^Si	2.61	20.1	79.9	0.0
ICP-SFMS in MR	0.08	4.3	38.8	56.9
ICP-QMS (reaction mode)	0.31	53.4	30.2	16.4
ICP-MS/MS	0.26	48.9	24.0	27.1
ICP-QMS (collision mode)	0.47	41.4	31.8	26.8

a Correction factor for masses.

b Fractionation factor.

## Abbreviations

MC: Multi-collector
SFMS: Sector field mass spectrometer
QMS: Quadrupole mass spectrometer
LOD: Limit of detection
BEC: Background equivalent concentration
IIF: Instrumental isotopic fractionation
IRMS: Isotope ratio mass spectrometry
VCDT: Vienna Canyon Diablo Troilite
eR: Edge mass resolution
*u*_c,rel_: Relative combined uncertainty
