# Supplementary material for: The performance of single and multi-collector ICP-MS instruments for fast and reliable 34S/32S isotope ratio measurements†
Source: Anal Methods. Author manuscript; Available in PMC 2016 Nov 14. (PMC5087850; doi:10.1039/C6AY02177H)
Supplement: Electronic supplementary information [file NIHMS70247-supplement-Electronic_supplementary_information.pdf]

Electronic Supplementary Material (ESI) for Analytical Methods.
This journal is © The Royal Society of Chemistry 2016

Table S1

1. Detailed operating parameters of the MC-ICP-MS (Nu Plasma HR)

RF power	1300 W
auxiliary gas flow rate	0.75 L min^{-1}
cool gas flow rate	13.0 L min^{-1}
lens settings	optimized for optimal sensitivity and peak shape
measurement statistics	6 blocks of 10 measurements
measurement time/sample	8 min
axial mass/mass separation	33/0.167; 29/0.125
lens voltages	quad 1 (S): -1.0 V to -2.1 V; quad 2 (S): 36.1 V to 37.8 V
	quad 1 (S); 27.3 V; quad 2 (Si): -67.8 V
	Cubic: -20; Q15: -16 (for (quasi)dynamic method)
detection system	Faraday collectors
resolution mode	edge mass resolution ($m/\Delta m$ = ~ 2700)*
	high mass resolution ($m/\Delta m$ = ~ 1900)
sample introduction system	*DSN 100 with PFA nebulizer*
sample uptake rate	100 – 120 µL min^{-1}
nebulizer pressure	30 – 40 psi
hot gas flow rate	0.08 L min^{-1}
membrane gas flow rate	3 - 4 L min^{-1}
spray chamber temperature	112 - 113°C
membrane temperature	122 - 123°C

* calculated according to [22]

2. Detailed operating parameters of ICP-SFMS (Element 2)

RF power	1300 W
sample gas flow rate	1.0 – 1.1 L min^{-1}
auxiliary gas flow rate	1.12 L min^{-1}
cool gas flow rate	16.0 L min^{-1}
lens settings	optimized for optimal sensitivity and peak shape
sample time	5 ms (^{32}S) and 10 ms (^{34}S)
mass window	20 %
sample time	0.005 (32S); 0.01 (34S)
samples per peak	80
search window	100 %
integration window	50 %
measurement statistics	20 passes and 50 runs
measurement time/sample	4 min
detection system	secondary electron multiplier (analogue mode)
mass resolution	$m/\Delta m$ = 4000
sample introduction system	*APEX-ACM, cooled cyclonic spray chamber*
sample uptake rate	100 – 120 µL min^{-1}
sweep gas flow rate	2 - 3 bar

3. Detailed operating parameters of ICP- QMS in reaction mode (NexION 350D)

RF power	1300 W
nebulizer gas flow rate	0.92 – 0.94 L min^{-1}
auxiliary gas flow rate	0.75 L min^{-1}
cool gas flow rate	15 L min^{-1}
lens voltage	optimized for optimal sensitivity
dwell time per amu	50 ms (32S16O+) and 200 ms (34S16O+)
measurement statistics	1 sweep, 600 readings, 1 replicate
measurement time/sample	4 min
detection system	secondary electron multiplier (pulse mode)
dead time	35 ns
cell gas flow rate	0.8 – 0.85 mL min-1 O2
RPq	0.40 – 0.50
axial field voltage	250 V
settling time	200 ms
sample introduction system	*Aridus II, cooled cyclonic spray chamber*
sample uptake rate	100 - 120 µL min^{-1}
N_2 gas flow rate	4 – 5 mL min^{-1}

Ar sweep gas flow rate	3 – 5 L min^{-1}

4. Detailed operating parameters of ICP-MS/MS (Agilent 8800)

RF power	1550 W
carrier gas flow rate	1.09 – 1.18 L min^{-1}
auxiliary gas flow rate	0.89 L min^{-1}
cool gas flow rate	15 L min^{-1}
lens voltage	optimized for optimal sensitivity
integration time	50 ms ($^{32}S^{16}O^+$) and 200 ms ($^{34}S^{16}O^+$)
measurement statistics	500 sweeps, 30 replicates
measurement time	2 min
detection system	secondary electron multiplier (pulse mode)
wait time offset	2 ms
dead time	31 ns
cell gas flow rate	0.30 mL min^{-1} O_2
settling time	100 ms
sample introduction system	*APEX-spiro TMD, cooled double-pass spray chamber*
sample uptake rate	150 - 200 µL min^{-1}
sweep gas flow rate	1.6 - 1.7 L min^{-1}

5. Detailed operating parameters of ICP-QMS/MS (Agilent 7500ce)

RF power	1600 W
carrier gas flow rate	1.25 L min^{-1}
auxiliary gas flow rate	0.9 L min^{-1}
cool gas flow rate	15 L min^{-1}
lens voltage	optimized for optimal sensitivity
integration time	50 ms (^{32}S) and 200 ms (^{34}S)
measurement statistics	3 sweeps, 100 replicates
measurement time	2 min
sample uptake rate	100 - 120 µL min^{-1}
detection system	secondary electron multiplier (pulse mode)
dead time	32 ns
cell gas flow rate	0.10 mL min^{-1} Xe
Qpole and Octopole bias	-20 V and -40 V
sample introduction system	*APEX-spiro TMD, cooled double-pass spray chamber*
sample uptake rate	1 mL min^{-1}
sweep gas flow rate	1.8 L min^{-1}

Table S2-1 Precision of ICP-MS instruments (*n* indicates the number of repeated measurements, IAEA-S-1, using IAEA-S-2 as bracketing standard)

Instrument / operation mode	repeatability (this work)	repeatability (literature values)
MC ICP-MS in eR		
**$^{34}S/^{32}S$ isotope ratio**		
eR (static)	0.003 % (*n* = 14)	0.01 % [37], 0.05 % [36], < 0.1 % [25]
HR	0.01 % (*n* = 14)	0.015 % [35]
eR (dynamic)	0.05 % (*n* = 16)	
eR (quasi-dynamic)	0.02 % (*n* = 15)	
**$^{30}Si/^{28}Si$ isotope ratio**		
eR (static)	0.003 % (*n* = 15)	
eR (dynamic)	0.08 % (*n* = 16)	
eR (quasi-dynamic)	0.03 % (*n* = 15)	
**$^{29}Si/^{28}Si$ isotope ratio**		
eR (static)	0.002 % (*n* = 15)	
eR (dynamic)	0.03 % (*n* = 16)	
eR (quasi-dynamic)	0.02 % (*n* = 10)	
**$^{30}Si/^{29}Si$ isotope ratio**		
eR (static)	0.003 % (*n* = 15)	
eR (dynamic)	0.07 % (*n* = 16)	
eR (quasi-dynamic)	0.02 % (*n* = 10)	
**$^{34}S/^{32}S$ isotope ratio**		
ICP-SFMS in MR	0.08 % (*n* = 14)	0.01 % [38], < 0.1 % [11, 20], 0.2 % [36], 0.4 % [37]
ICP-QMS		< 1.0 % [27]
ICP-QMS (reaction mode)	0.26 % (*n* = 15)	
ICP-MS/MS	0.24 % (*n* = 15)	
ICP-QMS (collision mode)	0.21 % (*n* = 18)	< 0.3 % [17]

Table S2-2 Within-lab reproducibility of ICP-MS isotope ratio measurements (*n* indicates the number of single measurements, IAEA-S-1, using IAEA-S-2 as bracketing standard). (HP stands for high performance skimmer cone)

Instrument / operation mode	reproducibility (this work)	reproducibility (literature values)
MC ICP-MS in eR		
**$^{34}S/^{32}S$ isotope ratio**		
eR	0.02 % (*n* = 20)	0.07 ‰ [23], 0.15 ‰ [8], 0.20 ‰ [10]
eR (dynamic)	0.20 %* (*n* = 12), 0.06 %** (*n* = 12)	
eR (dynamic) – HP cones	0.95 %* (*n* = 12), 0.19 %** (*n* = 12)	
eR (quasi-dynamic)	0.56 %* (n =16), 0.13 %** (*n* = 12)	
eR (quasi-dynamic) – HP cones	3.0 %* (*n* = 16), 1.2 %** (*n* = 16)	
**$^{30}Si/^{28}Si$ isotope ratio**		
eR	0.64 % (*n* = 17)	
eR (dynamic)	0.24 % (*n* = 12)	
eR (dynamic) – HP cones	1.09 % (*n* = 12)	
eR (quasi-dynamic)	0.62 % (*n* = 16)	
eR (quasi-dynamic) – HP cones	2.95 % (*n* = 16)	
**$^{29}Si/^{28}Si$ isotope ratio**		
eR	0.34 % (*n* = 17)	
eR (dynamic)	0.04 % (*n* = 12)	
eR (dynamic) – HP cones	0.11 % (*n* = 12)	
eR (quasi-dynamic)	0.13 % (*n* = 16)	
eR (quasi-dynamic) – HP cones	1.18 % (*n* = 16)	
**$^{30}Si/^{29}Si$ isotope ratio**		
eR	0.49 % (*n* = 17)	
eR (dynamic)	0.14 % (*n* = 12)	
eR (dynamic) – HP cones	0.60 % (*n* = 12)	
eR (quasi-dynamic)	0.38 % (*n* = 16)	

eR (quasi-dynamic) – HP cones	2.9 % (*n* = 16)	
**$^{34}S/^{32}S$ isotope ratio**		
ICP-SFMS in MR	0.21 % (*n* = 10)***	
ICP-QMS (reaction mode)	0.66 % (*n* = 50)	
ICP-MS/MS	0.27 % (*n* = 72)	
ICP-QMS (collision mode)	0.26 % (*n* = 64)	0.2 - 0.3 % [17]

* $^{34}S/^{32}S$ isotope ratio corrected for IIF with $^{30}Si/^{28}Si$ isotope ratio

** $^{34}S/^{32}S$ isotope ratio corrected for IIF with $^{29}Si/^{28}Si$ isotope ratio

*** calculated without setting of mass offset

Table S2-3 IIF per mass unit of ICP-MS instruments (percentage) (IAEA-S-1, using IAEA-S-2 as bracketing standard)

**Instrument / operation mode**	**IIF per mass unit (this work)**		**IIF per mass unit (literature values)**
MC ICP-MS in eR			
**$^{34}S/^{32}S$ isotope ratio**			
eR (static)	-4.05 - -4.78	(*n* = 26)	4.03 – 4.63 [36], 4.9 [12], 3.3 [25]
in 2 mg L^{-1} Na matrix	-4.01 - -4.08	(*n* = 3)	
in 2 mg L^{-1} Ca matrix	-4.16 - -4.24	(*n* = 3)	
in 2 mg L^{-1} Ca + Na matrix	-4.00 - -4.05	(*n* = 3)	
**$^{30}Si/^{28}Si$ isotope ratio**			
eR (static)	-3.64 - -4.52	(*n* = 25)	
in 2 mg L^{-1} Na matrix	-4.79 - -4.90	(*n* = 3)	
in 2 mg L^{-1} Ca matrix	-3.84 - -4.07	(*n* = 3)	
in 2 mg L^{-1} Ca + Na matrix	-4.31 - -4.70	(*n* = 3)	
**$^{29}Si/^{28}Si$ isotope ratio**			
eR (static)	-3.27 - -3.97	(n = 25)	
in 2 mg L^{-1} Na matrix	-4.27 - -4.30	(*n* = 3)	
in 2 mg L^{-1} Ca matrix	-4.26 - -4.30	(*n* = 3)	
in 2 mg L^{-1} Ca + Na matrix	-4.21 - -4.20	(*n* = 3)	
**$^{30}Si/^{29}Si$ isotope ratio**			
eR (static)	-4.34 - -5.13	(*n* = 25)	
in 2 mg L^{-1} Na matrix	-5.60 - -5.80	(*n* = 3)	
in 2 mg L^{-1} Ca matrix	-3.65 - -4.07	(*n* = 3)	
in 2 mg L^{-1} Ca + Na matrix	-4.69 - -5.48	(*n* = 3)	
**$^{34}S/^{32}S$ isotope ratio**			
ICP-SFMS in MR	0.03 – 0.48*	(*n* = 14)	0.7 [11], 2.92 – 3.04 [36]
ICP-QMS			< 1 % [27]
ICP-QMS (reaction mode)	-8.36 – 4.35	(*n* = 27)	

ICP-MS/MS	-5.84 – -2.24	(*n* = 37)
ICP-QMS (collision mode)	-8.78 – -5.81	(*n* = 30)

* calculated without setting of mass offset

Table S2-4 Deviation from the certified value (IAEA-S-1, using IAEA-S-2 as bracketing standard)

**Instruments / operation mode**	**deviation from certified value**
MC ICP-MS in eR	
**bracketing**	
static measurement mode	
eR	< 0.002 %* (*n* = 42)
HR	0.17 % - 0.31 % (*n* = 15)
**internal IIF correction – no correction of masses applied**	
dynamic measurement mode	
corrected with $^{30}Si/^{28}Si$	0.88 % - 0.97 % (*n* = 16)
corrected with $^{29}Si/^{28}Si$	0.86 % - 0.97 % (*n* = 16)
corrected with $^{30}Si/^{29}Si$	0.86 % - 0.97 % (*n* = 16)
quasi-dynamic measurement mode	
corrected with $^{30}Si/^{28}Si$	0.18 % - 0.51 % (*n* = 16)
corrected with $^{29}Si/^{28}Si$	0.24 % - 0.36 % (*n* = 16)
corrected with $^{30}Si/^{29}Si$	0.18 % - 0.52 % (*n* = 16)
**internal IIF correction – correction of masses applied**	
dynamic measurement mode	
corrected with $^{30}Si/^{28}Si$	< 0.002 %* - 0.02 % (*n* = 16)
corrected with $^{29}Si/^{28}Si$	< 0.002 %* - 0.02 % (*n* = 16)
corrected with $^{30}Si/^{29}Si$	< 0.002 %* - 0.02 % (*n* = 16)
quasi-dynamic measurement mode	
corrected with $^{30}Si/^{28}Si$	0.02 % - 0.09 % (*n* = 16)
corrected with $^{29}Si/^{28}Si$	0.02 % - 0.09 % (*n* = 16)
corrected with $^{30}Si/^{29}Si$	0.02 % - 0.09 % (*n* = 16)
ICP-SFMS in MR	0.01 % - 0.23 % (*n* = 14)
ICP-QMS (reaction mode)	-0.94 % - 1.1 % (*n* = 27)

ICP-MS/MS	-0.47 % - 0.41 % (*n* = 37)
ICP-QMS (collision mode)	-0.46 % - 0.36 % (*n* = 30)

* maximum significant deviation: 0.002 %
